# Climate Change and Genetic Structure of Leading Edge and Rear End Populations in a Northwards Shifting Marine Fish Species, the Corkwing Wrasse (*Symphodus melops*)

**DOI:** 10.1371/journal.pone.0067492

**Published:** 2013-06-26

**Authors:** Halvor Knutsen, Per Erik Jorde, Enrique Blanco Gonzalez, Joana Robalo, Jon Albretsen, Vitor Almada

**Affiliations:** 1 Institute of Marine Research (IMR), Flødevigen Marine Research Station, His, Norway; 2 Centre for Ecological and Evolutionary Synthesis (CEES), Department of Biology, University of Oslo, Oslo, Norway; 3 University of Agder, Kristiansand, Norway; 4 Unidade de Investigação em Eco-Etologia, ISPA-Instituto Universitário, Lisboa, Portugal; University of Connecticut, United States of America

## Abstract

One mechanism by which marine organisms may respond to climate shifts is range shifts. The corkwing wrasse (*Symphodus melops)* is a temperate fish species, inhabiting the coasts of Europe, that show strong indications of current as well as historical (ice-age) range shifts towards the north. Nine neutral microsatellite DNA markers were screened to study genetic signatures and spatial population structure over the entire geographic and thermal gradient of the species from Portugal to Norway. A major genetic break (*F*_ST_  = 0.159 average among pairs) was identified between Scandinavian and more southern populations, with a marked reduction (30% or more) in levels of genetic variability in Scandinavia. The break is probably related to bottleneck(s) associated with post-glacial colonization of the Scandinavian coasts, and indicates a lack of present gene flow across the North Sea. The lack of gene flow can most likely be attributed to the species’ need for rocky substrate for nesting and a relatively short pelagic larval phase, limiting dispersal by ocean currents. These findings demonstrate that long-distance dispersal may be severely limited in the corkwing wrasse, and that successful range-shifts following present climate change may be problematic for this and other species with limited dispersal abilities, even in the seemingly continuous marine environment.

## Introduction

Currently, climate change is one of the major factors reducing the abundance of many marine species, and increasing the likelihood of local extinction in the oceans [1], [2]. Temperature controls the rate of fundamental processes, and thereby regulates organism attributes, including developmental rate and survival [3]. Because many marine organisms already live close to their thermal tolerances [4], increases in temperature can negatively impact their performance and survival. The increase of temperature during the last few decades in marine ecosystems around the world [5], has substantiated climate warming. At present, the situation e.g. in the North Atlantic and Mediterranean Ocean is such that if warming occurs according to what is predicted globally, we are to expect local extinctions of species less adapted to higher temperatures in the Mediterranean and in Southwest Europe, as well as northward shift in species’ distribution. During the last decades, both types of shifts have been documented, with warm-water organisms being increasingly recorded in areas to the north of their usual range, and local extinction or decrease in abundance in cold temperate species in the south [6]–[8].

In order to predict potential future impact on marine organisms due to the current global climate change, we may study the consequences of earlier, large-scale climate shifts, i.e. those associated with the most recent glacial period. During glacial phases, the sea surface temperatures along western Europe dropped markedly, and the majority of warm temperate organisms must have moved south, either into the Mediterranean, along the West-African shores, or in glacial refugia in warm-water pockets closer to the glaciated regions [9], [10]. After the last glacial maximum (LGM; 17–20 kBP), and especially at about 10 000 BP, temperatures were rising markedly, leading to a rapid meltdown of the glaciations in the north that flooded the North Sea at about 8000 BP [11]. With the reestablishment of interglacial conditions, marine species ranged along the stretch of the coast comprised between the Mediterranean and the Baltic. Depending on their thermal tolerances, warm water species have their northern limits along this latitudinal gradient, while cold temperate species have their southern limits along that same gradient.

The generally wider distribution ranges occupied by marine species compared to terrestrial species has been suggested to produce a buffering effect against rapid fluctuation in the environmental conditions, lowering the risk of extinction in the former group [6], [12]. However, a large number of marine organisms from different taxonomic groups are in decline or have shifted their distribution ranges in response to variations in the environmental conditions [7], [8]. The geographical extent of the expansion/contraction events depends on the combination of intrinsic biological factors related to the species’ life history and its physiological optimum and the magnitude and duration of the new environmental conditions. Depth acts as limiting factor in the distribution and dispersal of marine organisms, as altitude does in the terrestrial ecosystem [6]. While the establishment of warmer sea surface temperatures after the LGM favoured northwards colonization by warm temperate species from the Eastern North Atlantic [6], it also led to a raise in the sea level (up to 120 m) and associated large-scale changes in the coastline [11], that could have a major impact on both aquatic and terrestrial demographic and evolutionary processes.

Range shifts, population bottlenecks, and expansions are demographic processes that may lead to particular genetic signatures of extant populations [13], [14]. Hence, current patterns of genetic diversity should not be ascribed exclusively to contemporary processes, but also to historical episodes such as the oscillations in paleoclimatic conditions that altered oceanographic conditions and likely gene flow dynamics [7], [10], [15]. Successive expansion and contraction events occurring at leading edges and rear ends of marine species’ distributions, as response to climatic oscillations, may result in population bottleneck and reductions in intraspecific genetic diversity [7], [9], [16], [17]. In this regard, rapid northward post-glacial colonization likely prompted genetic homogeneity across extensive areas in Eastern North Atlantic temperate species, whereas slow dispersal probably tended to preserve local gene pools. On the other hand, contraction episodes appear associated to population shrinks and selective forces, reducing effective sizes and eroding the genetic resources [6], [7], [17]. Additionally, the topography would condition species dispersal and gene flow [6], [18].

**Figure 1:**
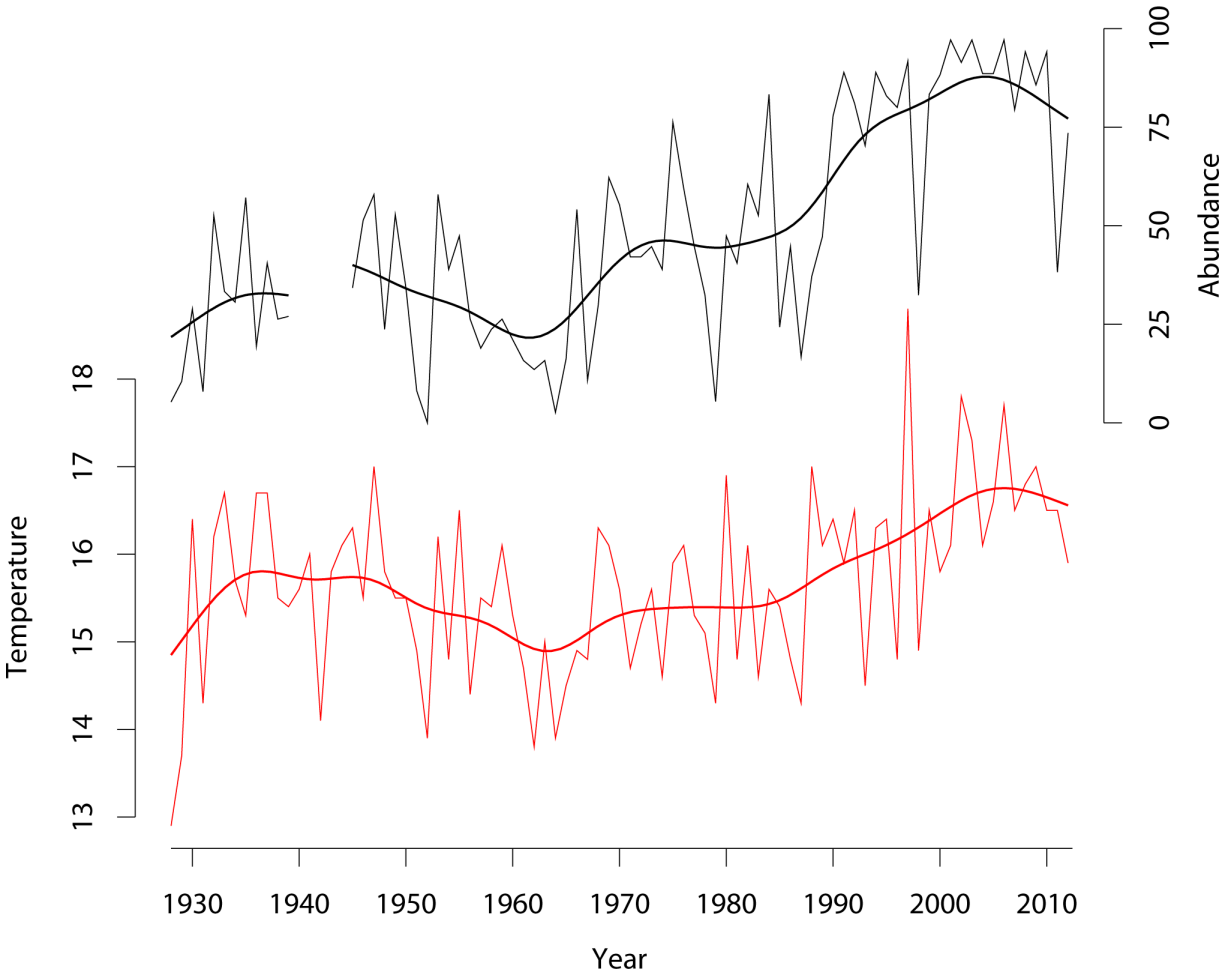
Average water temperature during summer (red) and percentage occurrence of corkwing wrasse (black) in beach seine hauls in Skagerrak from 1928 to 2012, based on annual measurements by the Institute of Marine Research [61]. Solid lines represent kernel smoothed averages (using function ksmooth of the R statistical package [62]).

Corkwing wrasse (*Symphodus melops,* Linnaeus, 1758*)* is a rocky shore marine fish suspected to have shifted northwards in abundance over the last few decades. In its southern range (“rear edge”) it is now nearly extinct in the Mediterranean [19]. At the same time, it has become increasingly more common in the northern areas, e.g., along the Norwegian Skagerrak coast, where abundances of coastal fishes have been monitored on an annual basis since the early 20’th century (Fig.1). A recent study of the mitochondrial control region and the first intron of the nuclear S7 ribosomal protein gene found a significant reduction in genetic diversity, which was associated to a northward recent expansion [20]. In the present study we expand on this work and include more sample locations, larger samples, and several more nuclear (microsatellite) markers, in order to elucidate genetic footprints of past and present range-shifts and resolve the colonization route into Scandinavia. We further include oceanographic modelling to characterize the species’ dispersal abilities at the pelagic larval stage and interpret genetic patterns in light of present and historical patterns and processes.

## Materials and Methods

### The Species Studied

The Labridae family is the third largest family of marine fish, with 580 species in 82 genera [21]. The corkwing wrasse belongs to this family and is a rocky shore species, inhabiting temperate Atlantic waters, and whose abundance has been correlated to water temperatures above 7°C [22]. The species may reach an age of 9 years and about 28 cm in length. Males grow faster than females. Female-mimicking sneaker males are sometimes observed (3–20% of all males) with slower growth [23]. This species is believed to be non-migratory with territorial behaviour, inhabits shallow coastal water, and is most commonly found in the upper 30 m of the water column. Males build and guard seaweed nests among rocks or in crevices, and ripe females show a short ovipositor during summer. The species presents a pelagic larval phase probably lasting between 14–17 days (results of MarinEra project, Borges pers com.) and 25 days [22]. The corkwing wrasse is presently distributed along the European coasts, from southern Portugal to western Norway except along the sandy coast between the Netherlands and Denmark. Earlier, it was also frequently found in Mediterranean waters, but has become extremely rare, probably due to an increase in temperature [19]. Now the species is found scarcely in southern Portugal, with single individuals occasionally appearing at Azores islands (Stefanni, pers. Com.). In contrast, the species is becoming more abundant in Norway, according to the long term beach seine monitoring program carried out by the Institute of Marine Research, Norway (Fig. 1).

### The Study Area

**Figure 2:**
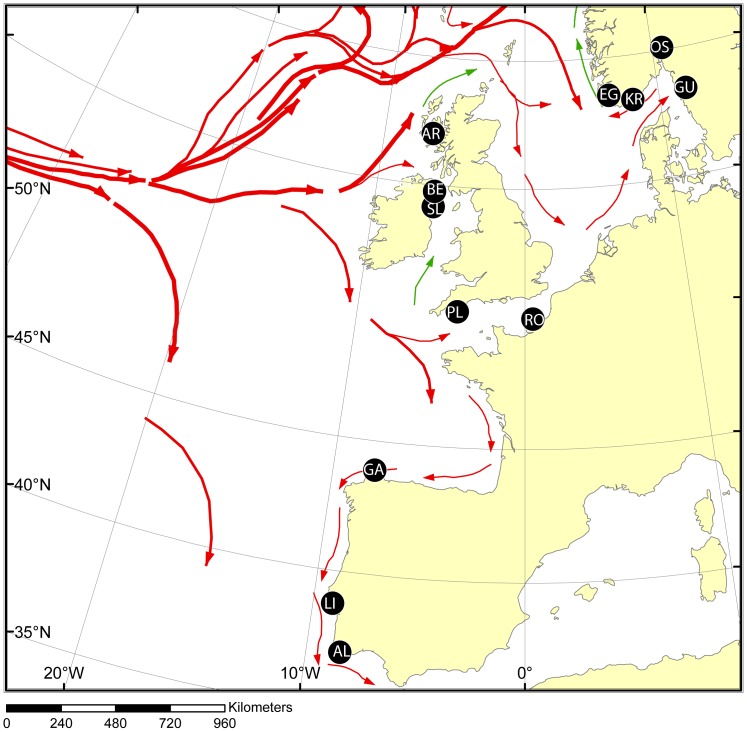
Map identifying sample locations (black circles: see Table 1 for sample abbreviations and details). Red arrows denote the predominant surface currents relevant for the pelagic (larvae) face of the cork wing wrasse.

The study area spanned across the species distribution range from southern Portugal (Algarve) to Scandinavia (cf. Fig. 2). The area varies greatly from south to north with respect to several parameters such as wave exposure, temperature, and tidal amplitude [24] (see webpage of Gyory et al, for details: http://oceancurrents.rsmas.miami.edu/atlantic/atlantic.html). Briefly, the coastline in the south is fully exposed to the Atlantic Ocean with very high tidal amplitude and with strong coastal currents and waves, and temperature is at the maximum of the species tolerance. In the north (Scandinavia) temperature is at the cold tolerance limit [25], and tidal amplitude is nearly absent and exposure to waves and coastal currents are less pronounced due to numerous skerries, bays, and fjords breaking waves down. The central distribution area in the UK and Ireland is more of an intermediate between the north and the south: rocky shore is of medium tide amplitude and occurs partly in sheltered loughs or bays. Interestingly, the sandy habitat prevalent from the Netherlands to Denmark (with the possible exception of Helgoland island), seems unable to hold viable populations of the species, probably due to its preference for rocky substrate.

### Sampling

Samples of *S. melops* were collected using various gears: beach seine in Norway and Sweden [22]; fish pots in UK; and spear fishing in Portugal and Spain [20]. Seine- and pot-sampled fish were immediately transferred to a sea water tank and sacrificed in the most gentle and swift way by percussive stunning with a priest, in accordance with relevant legislation in each country (Norway: Dyrevelferdsloven §12 (law of animal welfare: http://www.lovdata.no. Accessed 2013 Jun 3.); Sweden: Djurskyddslagen §13 (animal protection law: http://www.notisum.se/. Accessed 2013 Jun 3.); UK: Animal Welfare Act 2006 (http://www.legislation.gov.uk. Accessed 2013 Jun 3.)). Spear fishing (Portugal and Spain) was carried out by divers with a permit (#246/2000). All sampling and handling of fish were performed by experienced personnel (by scientists in Norway and Portugal and by commercial fishers in Sweden and the UK), and were carried out explicitly for this study. The species is not protected by European law or by any of the countries where and when sampling was performed (it is a commercially harvested species in Scandinavia and Scotland and used as a “cleaner” fish in the salmon aquaculture industry), and no special permits are required for sampling the species either for research or commercially. Whole sacrificed fish were immediately frozen and stored (either at −20C or −70C on dry ice) until reaching laboratory facilities, where tissue was taken and put into 96% ethanol prior to DNA extraction.

### Genetic Analysis

DNA was extracted using the Viogene Inc. extraction kit (Sunnyvale, CA). Microsatellite DNA fragments were separated using a 16 capillary ABI 3170 automated sequencer. We applied Qiagen 5 Taq DNA Polymerase for the PCR reactions, using the supplied self-adjusting magnesium 10× buffer. PCR analyses were based on nine microsatellite DNA loci, using published protocols [26]. As a guard against potential genotyping errors, all capillary traces where scored independently by two trained persons using the software GeneMapper v. 4.0 (Applied Biosystems) In case of disagreement, a new PCR reaction was performed and individual genotypes re-scored We applied Microchecker software [27] to check for the presence of null alleles or other problems, but found none (data not shown).

### Statistical Analyses

The amount of genetic variability was characterized by heterozygosity or gene diversity (*Hs* within samples; *H*_T_ for the total [28]), and by observed number of alleles and allelic richness in the FSTAT software [29]. Deviations from Hardy-Weinberg genotype proportions were estimated by *F*_IS_ [30] and assessed using the exact probability test in the genepop software (ver. 4.0: [31]). Here, and in subsequent situations of multiple tests, we adopted the False Discovery Rate approach (FDR) [32] when interpreting test significances.

Genetic differences among localities were quantified by *F_ST_*, using Weir & Cockerham’s [30] estimator *θ* over all samples and between pairs of sample localities. Allele frequency differences among localities were tested for using exact tests in the genepop software [31] with 10 000 dememorizations and batches, using 10 000 iterations per batch. The reported *P*-values (summed over loci with Fisher’s summation procedure) were used to judge “significance” of estimated *F*_ST_-values, using the FDR approach to control for type I statistical errors in multiple tests. Temporal stability of spatial structure was tested for with AMOVA analyses of spatial samples and temporal replicates, using Arlequin ver. 3.5 [33]. This analysis was performed only with samples from Norway and the Iberian Peninsula due to the absence of temporal replicates from the UK. Because of the genetic “break” between UK and Norway (cf. results), we performed separate AMOVA analyses on each side of this barrier. To visualize spatial genetic differentiation patterns we applied a Principal Component Analysis (PCA) based upon allele frequencies, using the software PCAGEN (vers. 1.2.1) [34] and a Bayesian clustering method implemented in the software STRUCTURE v. 2.3.3 [35]. For STRUCTURE we used 7 repetitions for each value of K, for K 1–7 subpopulations, with Markov chain Monte Carlo resampling using 100 000 repetitions after a burn-in of 200 000.

The phylogenetic relationship among populations was examined by constructing a hybrid tree following Angers and Bernatchez [36]. Initially, Cavalli-Sforza and Edwards’ chord distance (*D*_CE_) [37] and Goldstein et al.’s δµ^2^ [38] were calculated with Populations (ver. 1.2.28) [39]. *D*_CE_ distance has been suggested to achieve better estimates of tree topology compared to other methods, regardless of mutation model, IAM or SMM [40]. On the other hand, SMM based δµ^2^ has been proposed to estimate branch lengths. The tree topology was therefore determined by a neighbour-joining (NJ) tree using the matrix of *D*_CE_ distances with PHYLIP [41], performing 1000 bootstraps. Finally, the hybrid tree was built selecting the user tree option in the Fitch program of PHYLIP [41], imposing the *D*_CE_ tree topology and determining the branch lengths from Goldstein et al.’s δµ^2^ matrix [38] using Cavalli-Sforza and Edwards’ least-square method [37].

In order to clarify possible dispersal and/or colonization routes into Scandinavia, two additional analyses were conducted. Firstly, we performed an assignment tests (Geneclass 2.0 software; [42] using a Bayesian approach to elucidate if the most likely invasion route was through the English Channel or by way of the north UK. Here, the three northerly UK samples (SL, BE and AR), and the two southerly samples close to the entrance of the English Channel (PL and RO) were grouped and defined as the two baseline “samples”, whereas the four Scandinavian samples were used as “samples to be assigned” (cf. Table 1 for explanations of abbreviations). The reason for grouping samples is to reduce statistical noise in the data that increases rapidly in assignment analysis with more samples included.

**Table 1:** Summary information on corkwing wrasse samples, including information on abbreviated names (Abbrev.), sample size for each of the years (2008–2010), and geographical position (Lat = latitude and Long = longitude, in decimal degrees).

			Samples (n)	Position	
Sample location	Abbrev.	Region	2008,2009,2010	Lat	Long
Gulmarsfjord	GU	Scandinavia	50,46,0	N 58.18	E 11.32
Oslo	OS	Scandinavia	50,46,0	N 59.52	E 10.39
Kristiansand	KR	Scandinavia	25,50,21	N 58.11	E 8.34
Egersund	EG	Scandinavia	23,38,0	N 57.27	E 5.53
Ardtoe	AR	UK	0,0,96	N 56.4	W 5.50
Strangeford Lough	SL	UK	0,0,75	N 54.49	E 5.46
Belfast	BE	UK	0,21,0	N 54.33	W 5.46
Plymouth	PL	UK	0,72,0	N 50.34	W 4.45
Roscoff	RO	France	0,23,0	N 48.46	W 3.58
Galicia	GA	Iberian Peninsula	0,60,36	N 42.18	W 8.81
Lisbon	LI	Iberian Peninsula	0,39,55	N 38.83	W 9.47
Algarve	AL	Iberian Peninsula	0,29,67	N 37.12	W 8.53

Secondly, we performed oceanographic modelling to check if transport of pelagic larvae from around the UK towards the Skagerrak is possible under the present ocean currents regime and given a maximum pelagic larval duration of 25 days. Both the regional ocean circulation model (ROMS) and the particle-tracking model conducted are similar to the model systems described by Vikebø et al. [43], and technical details can be found there. Briefly, we released 1800 virtual particles close to some of the sample locations, and additionally introduced two fictive spawning locations near the Orkneys north of UK and off Lowestoft (UK) in the southern North Sea. The two fictive locations were chosen due to their relative adjacency to the Skagerrak. Instead of releasing all particles at one single point, they were spread over an area of approx. 8×8 km. Because the ocean current model is limited in resolution (4 km in the horizontal), the particles were released off the coast in open sea. Our drift simulations then consider egg-particles drifting offshore only, and as we considered the maximum pelagic larval duration, the estimated drift pathways may be considered a maximum. Each individual particle was held at a fixed depth during the tracking simulations, and to represent all potential drifting depths, the initial depth of each particle was found randomly with mean and standard deviation of 10 and 5 m, respectively. The experiment was repeated, using realistic, physical summer conditions (ocean currents for June) for all years from 2000 to 2011, i.e. 12 drift experiments, to capture the year-to-year variability of the oceanic conditions. Additional particles were released from two of the Norwegian sample locations to access the potential for spread within Scandinavian waters and to the surroundings.

## Results

The results are based on a total of 922 individual corkwing wrasse (Table 1), scored at nine microsatellite loci each (Table 2). The loci generally conformed to Hardy-Weinberg genotype expectations (cf. Table 3). Of the 9*12 = 108 different locus-sample combinations, 14 were statistically significant at the 5% level, and five of these remained significant under the FDR approach. We found no apparent pattern to these departures from Hardy-Weinberg genotype proportions, and *F*_IS_ estimates and *P*-values varied among loci within samples as well as among samples at the same locus (cf. Table 3).

**Table 2:** Amount of genetic variability among corkwing wrasse samples at nine microsatellite loci.

Locus	*a*	*H_T_*	*F_ST_*
SMB11	90	0.925	0.1295***
SMD112	27	0.855	0.0651***
SMD110	27	0.844	0.0807***
SMC8	35	0.912	0.0710***
SMA103	12	0.741	0.0701***
SMA11	18	0.636	0.0609***
SMD121	43	0.901	0.0526***
SMD131	26	0.829	0.1045***
SMA107	13	0.569	0.1386***
Average	32.3	0.801	0.0848***

*a* is the observed number of alleles; *H*_T_ [28] is the gene diversity in the total material (n  = 922); *F*_ST_ [30] estimates the level of genetic differentiation among the twelve samples, with statistical support indicated by exact test for allele frequency heterogeneity (GENEPOP v. 4.0.6: [31]: *** *P*<0.001).

**Table 3:** Summary statistics for genetic variability within sample sites.

Sample location	*H*_S_	*a*	*A*_r_	*F*_IS_	*P*-value	N loci excess	N loci deficiency
GU	0.563	8.3±3.7	5.3±2.3	−0.0047	0.230	7	2
OS	0.576	8.1±3.9	5.0±2.0	−0.0074	0.087	3	6
KR	0.597	8.1±3.4	5.4±2.0	0.0561	0.001	2	7SMD112, SMC8, SMA103
EG	0.574	7.5±4.5	5.3±2.5	0.0056	0.940	6	3
AR	0.839	20.6±15.3	11.5±5.4	−**0.0279**	0.001	3SMA107	6SMA103
SL	0.808	16.6±10.8	10.6±5.1	**0.0267**	0.001	3	6
BE	0.817	11.2±5.0	10.6±4.7	0.0779	0.051	4	5SMB11, SMA11
PL	0.827	17.8±10.0	11.0±5.1	0.0041	0.420	2	7
RO	0.833	12.4±5.8	11.0±4.9	0.0021	0.380	4	5
GA	0.830	20.7±13.7	11.2±5.1	**0.0063**	0.001	4SMA107	5SMB11, SMA103
LI	0.816	20.3±13.5	11.0±5.2	−**0.0039**	0.001	5SMD110, SMD131	4SMD121
AL	0.828	19.7±12.3	11.1±5.0	−**0.0315**	0.001	5SMA107	4
							
Average	0.742	15.3.±2.84	9.1±4.9	0.0110	0.001	4	5

*H*_S_ is the estimated average gene diversity [28]; ***a*** is the average number of alleles per locus, and *A*r is the average allele richness ±1X standard deviation. *F*_IS_ refer to average deviations from Hardy-Weinberg genotype proportions, with number of loci deviating in either direction given. P-values refer to the overall two sided test over all loci (Fisher’s summation procedure over nine single-locus tests), and locus names identify the tests that came out significant (no adjustments made for multiple tests). Bold values of *F*_IS_ denote significant values in either direction.

**Figure 3:**
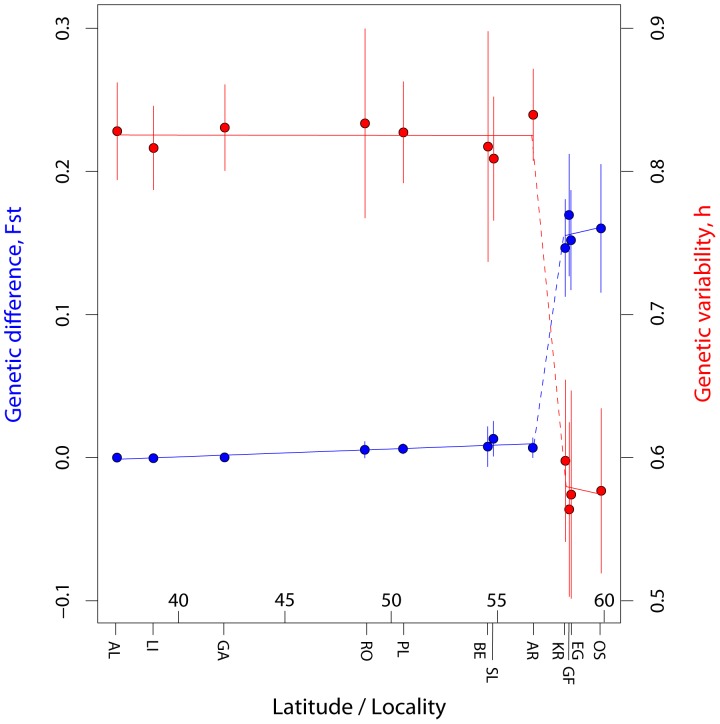
The figure illustrate the cline of *F*_ST_ (blue) and expected heterozygosity (red) with latitude. Lines connecting points are linear regression lines, calculated separately for the southern and northern (Scandinavian) parts, with dotted lines indicating the "genetic break" across the North Sea. The 95% confidence intervals are indicated with vertical bars for each point estimate, calculated as: mean +/−1.96*SE (i.e., assuming normal distribution). For *F*_ST_ standard errors were calculated by jackknifing over loci,; for heterozygosity (h), standard errors were taken as the square root of the intralocus variances, averaged over loci (equations 8.12 and 8.14 of ref. [63]).

**Figure 4:**
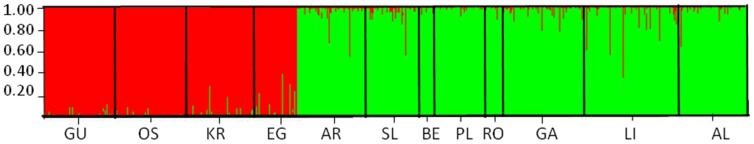
Principal Component Analysis on allele frequencies [34]. Note the differences in variability explained by the two axes (approx. 88% and 3% respectively).

**Figure 5:**
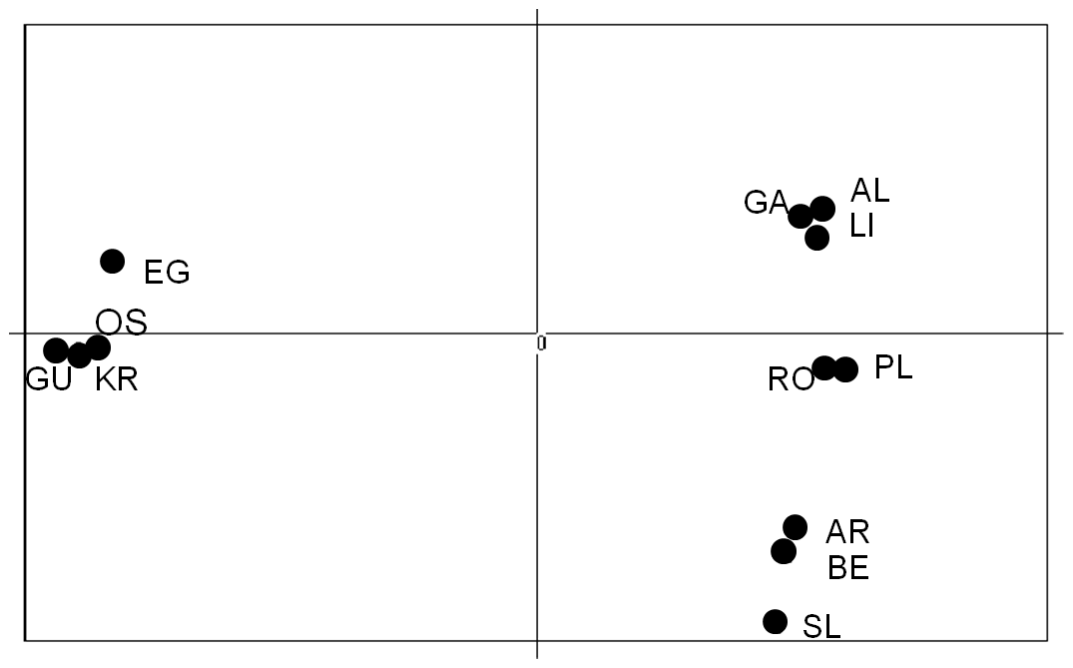
Bayesian clustering analysis of corkwing wrasse (*S. melops*) detected by STRUCTURE. A K = 2 was the most likely outcome.

Genetic variability (heterozygosity) differed among loci and averages over sample sites ranged from *H*_T_  = 0.569 (SMA107) to 0.912 (SMC8), while the total number of alleles spanned from *a*  = 13 (SMA107) to 90 (SMB11) (Table 2). Genetic variability varied among samples and both expected heterozygosity and allelic richness were higher among Atlantic samples (average *H_S_*  = 0.83, *A*_r_  = 11.0), as compared to the Scandinavian ones (*H_s_*  = 0.57, *A*_r_  = 5.2). With the exception of two singletons (at SMA11 and SMD110), no alleles were observed in the Scandinavian samples that were not also found in samples to the south. Viewed as a function of latitude, these differences among samples in genetic variability measures may be seen as an abrupt decline in variability (heterozugosity) at around 57 to 58 °N (i.e., the North Sea. This abrupt decline in heterozygosity on each side of the North Sea was also significant (t test gives t = 31.03; P<<0.0001), and accompanied by an equally abrupt increase in genetic differentiation (*F*_ST_; Fig. 3). The PCA plot (Fig. 4) identifies the separation of Scandinavian and Atlantic samples along the 1. principal axis, and splits the Atlantic samples roughly according to latitude along the 2. axis. This genetic break in the North Sea, which was shared among all loci (cf. Table 2), is a dominant feature of the data and was also the main finding using the software STRUCTURE (cf. Fig. 5). Below we present additional characteristics of Scandinavian and Atlantic samples separately.

Overall genetic differentiation was high and statistically significant (*F*_ST_  = 0.085, *P*<0.001 cf. Table 2), though largely due to pair-wise estimates between Skagerrak and Atlantic samples, ranging from 0.137 and 0.194 (cf. Table 4). Among Atlantic samples, pairwise *F*_ST_-estimates (Table 4) were generally low, and many of them were not statistically different from zero, as judged by allele frequency heterogeneity tests. In particular, we found no divergence (average *F*_ST_ = −0.0001; with a 95% Confidence Interval of −0.0009 to 0.0006, based on jackknife over loci) among the three southernmost samples from the Iberian Peninsula (AL, LI, and GA), whereas samples from France and the UK (RO, PL, BE, SL, and AR) for the most part were differentiated from each other (average *F*_ST_  = 0.0064; 95% CI  = 0.0018 to 0.0100), as were samples from Scandinavia (GU, EG, KR, and OS) (average *F*_ST_  = 0.0054; 95% CI  = 0.0017 to 0.0087). AMOVA analyses (Table 5) revealed that temporal differences (i.e., sample year) were lower (and not statistical significant) than spatial differences (sample locality), both overall and separately within each side of the North Sea genetic barrier.

**Table 4:** Genetic differentiation (*F*_ST_) between all pairs of sample localities.

	AL	LI	GA	RO	PL	BE	SL	AR	GU	EG	KR
AL											
LI	−0.0004										
GA	0.0001	−0.0000									
RO	***0.0058***	***0.0054***	0.0023								
PL	***0.0061***	***0.0044***	***0.0052***	***0.0050***							
BE	***0.0078***	***0.0061***	***0.0063***	0.0064	***0.0077***						
SL	***0.0127***	***0.0115***	***0.0119***	***0.0091***	***0.0116***	−0.0034					
AR	***0.0066***	***0.0066***	***0.0071***	***0.0067***	***0.0073***	0.0033	***0.0038***				
GU	***0.1700***	***0.1632***	***0.1594***	***0.1938***	***0.1833***	***0.1794***	***0.1640***	***0.1572***			
EG	***0.1523***	***0.1467***	***0.1427***	***0.1752***	***0.1654***	***0.1612***	***0.1451***	***0.1404***	−0.0013		
KR	***0.1469***	***0.1418***	***0.1368***	***0.1709***	***0.1601***	***0.1556***	***0.1446***	***0.1366***	***0.0106***	***0.0037***	
OS	***0.1603***	***0.1531***	***0.1497***	***0.1844***	***0.1702***	***0.1636***	***0.1526***	***0.1479***	0.0040	***0.0039***	***0.0075***

Statistical significant (at the 5% level or better, applying the False Discovery Rate approach) tests for allele frequency heterogeneity over loci are indicated with bold-italics font.

**Table 5:** Results of AMOVA analysis [33] partitioning genetic variability among and within localities (temporal replicates) within the Scandinavian and the Iberian regions.

		*F* - index	Variance	% of total	*P*-value
Scandinavia					
	Among samplelocalities (*F*_CT_)	0.0058	0.012	0.46	0.03*
	Among years within localities (*F*_SC_)	0.0017	0.004	0.17	0.22
Iberian Peninsula					
	Among samplelocalities (*F*_CT_)	−0.0004	−0.002	−0.04	0.33
	Among years within localities (*F*_SC_)	0.0006	0.002	0.06	0.25

* *P*<0.05.

**Figure 6:**
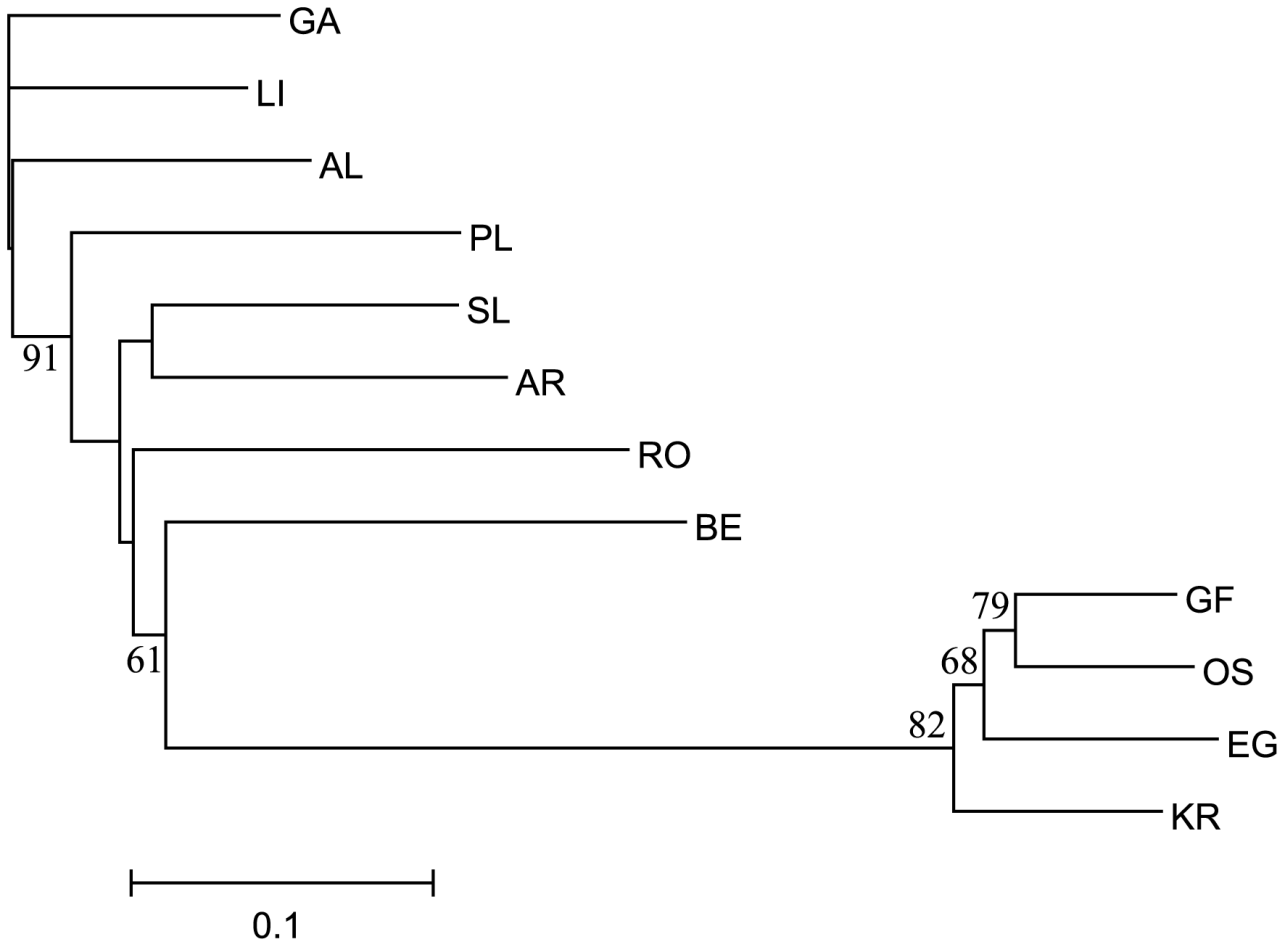
Phylogenetic relationship between *S. melops* populations. The tree topology was estimated from Cavalli-Sforza and Edward`s chord distance [37], *D*_CE,_ whereas the branch lengths were extracted from Goldstein et al.’s δµ^2^ distance matrix [38]. Numbers next to branch are bootstrap support above 60% (1 000 replicates).

The phylogenetic relationship among corkwing wrasse populations was resolved by the hybrid tree, which differentiated three apparent clusters with relatively high (over 60%) bootstrap support (Fig. 6). Each cluster corresponded to populations from the same geographic region, i.e., Iberian Peninsula, UK-France and Scandinavia. The branch length separating the cluster of corkwing wrasse from Iberian Peninsula and UK-France was much shorter than the one separating them from Scandinavian samples. The nodes among Scandinavian samples also presented bootstrap support above 60% (68–82%), although branch lengths were relatively short.

Assignment tests revealed that more than 92% of individuals from the four Scandinavian samples were assigned to the northern UK samples (SL, BE and AR lumped), rather than to the southern baseline group (PL and RO) (cf. Table 6). We also applied a subset of random individuals from west UK, in order to analyse equal number of individuals in the two groups, giving nearly identical results (not shown).

**Table 6:** Results of statistical assignment tests [42] over the North Sea genetic break.

	UK North	UK South
Egersund	100	0
Kristiansand	95.8	3.2
Oslo	93.8	5.8
Gulmarsfjord	95.8	3.2

Numbers denote the percentage of fish from each of the four Skagerrak samples being assigned to either the UK North (lumping AR, BE and SL) or the UK South (lumping PL and RO).

**Figure 7:**
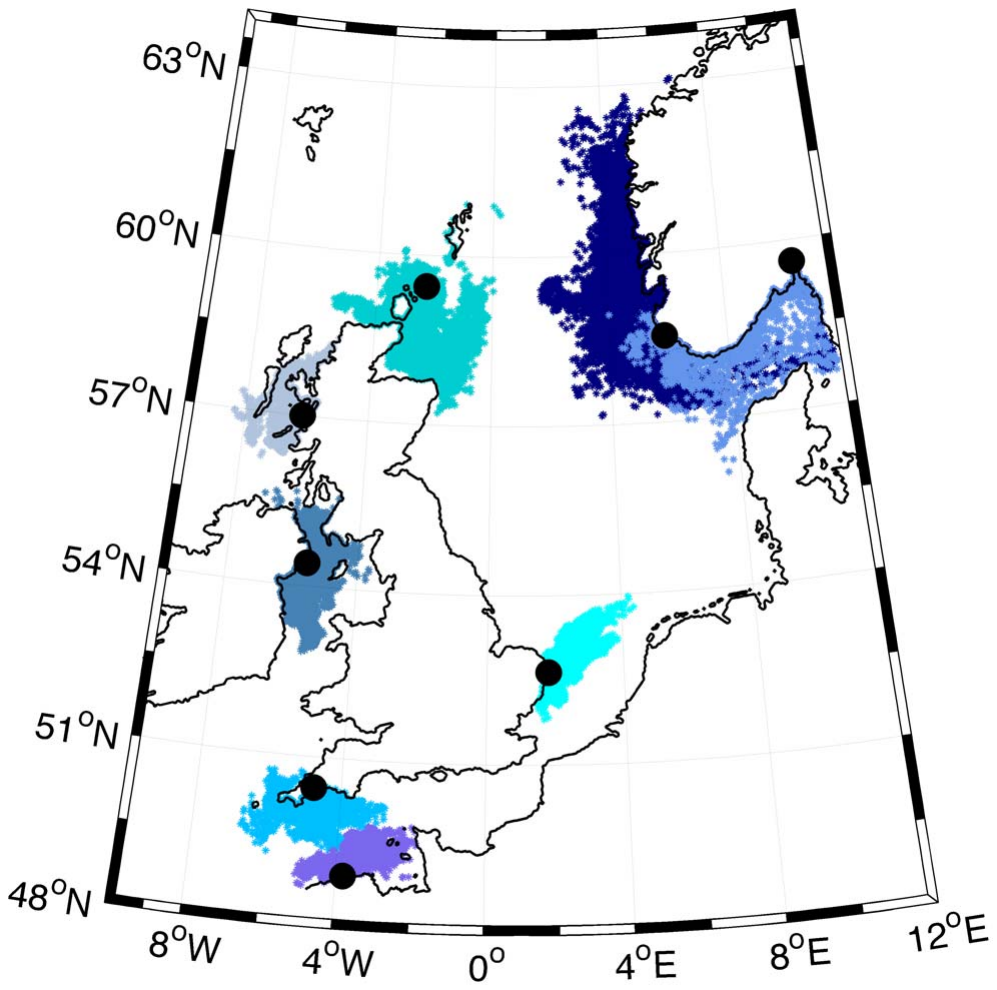
Modeled oceanographic drift of particles released at some of the sample locations (see positions in Fig. 2) in addition to two fictive UK locations (one near the Orkneys and one in the southern North Sea). The black filled circles denote the location where particles are released, and the colored clouds of dots denote where the particles end up after 25 days. Drift during a summer period (June) from 12 subsequent years (2000–2011) is displayed in this figure. Note that all particles are released offshore due to coarse resolution in the ocean current model. Our drift estimates then represent a maximum extension of the drift pathways.

Oceanographic drift simulations (Fig. 7) show that transport of pelagic larvae from the UK, across the North Sea and into the Skagerrak, is highly unlikely to happen during one season, as is the reverse transport from Skagerrak to the UK. The pelagic phase is too short according to net drift within the North Sea. For particles drifting offshore along the Norwegian Skagerrak coast, the relatively strong Norwegian Coastal Current may introduce a high degree of dispersal, mainly west- and northward along the coast.

## Discussion

Our findings support and elaborate on those of Robalo et al. [20], by identifying a major genetic discontinuity or break between the Scandinavian and the Atlantic (i.e., southern) samples. The large *F*_ST_ values (cf. Table 4) observed across the North Sea must be considered exceptionally high for a marine fish species (cf. refs 44,45). Additionally, the present study provides insight into a number of issues not previously addressed: 1) We document (Fig.1) a marked increase in abundance of the species in Scandinavian waters since the 1980’s, when temperatures started rising, and it seems likely that it is currently undergoing a range shift northwards. 2) By contrasting the particle transport simulation with the genetic results, we find that the lack of suitable habitat over some distance may have prevented population connectivity within the species. 3) Our results suggest that the invasion route between the UK and Scandinavia probably occurred along the predominant oceanic transport route west and north of UK rather than through the English Channel. 4) We report statistical significant evidence for genetic structure at fine geographical scale, among samples in Scandinavia and within UK. Below we discuss these findings in detail and their possible general implications.

The major genetic break over the North Sea was associated with a similar discontinuity in the levels of genetic variability, with reduced levels in the north. Relative to the southern region, the reduction in heterozygosity in Scandinavia is approximately 1–0.58/0.83 = 0.30 or 30%, and the reduction in the number of alleles is even greater (average of 8 vs.17.4 being 54% lower). Such a decline is expected in a severe bottleneck over a brief time period, or of less severe reductions in effective population size over a longer period. Robalo et al. [20] attempted to age the reduction in Scandinavian mtDNA variability in this species, and arrived at a number of estimates (under different mutation rate assumptions) that were all consistent with a post-glacial date for the bottleneck (point estimates varied from 1130 years BP to 3200 years BP for assumed mutation rates of 3.5 to 10% per million years, respectively, but with wide confidence intervals in any case).

The presumed Scandinavian bottleneck was also reflected in the phylogenetic tree (Fig. 6), whose major feature may be seen in the exceptionally long branch separating contemporary Scandinavian populations from two additional clusters corresponding to older mid- and low-latitude populations from UK France and Iberian Peninsula, respectively. Genetic differentiation between the latter two geographical regions was supported by high bootstrapping values (91%); nevertheless, they presented shorter genetic distances, probably reflecting recent and perhaps on-going genetic exchange.

On a finer geographic scale, genetic divergence among samples within regions (France-UK and Scandinavia), is expressed by significant pairwise allele frequency differences (Table 4) and high bootstrapping support among shorter phylogenetic branches (Fig. 6), showing a tendency for the species to substructure into local populations. Such local populations have been documented previously among Scandinavian [46], [47] and British [48]–[50] coastal fishes, and may be attributed to local topography (sheltered bays, fjords etc.), favouring local spawning and larval retention, generating population structure. The apparent absence of such structuring within the Iberian Peninsula, in contrast, may result from corkwing wrasse larvae in this region being exposed to stronger currents and higher dispersal, leading to higher gene flow, as observed in other species in that region [13], [51], [52].

On the basis of the findings reported herein and those by Robalo et al. [20], it seems clear that post-glacial colonization of Scandinavia by the corkwing wrasse was associated with a severe restriction in effective population size, i.e. a genetic bottleneck. Scandinavian populations still lack genetic variability compared to their more southern conspecifics, and the level of genetic differentiation across the North Sea (*F*_ST_  = 0.159, approx: Table 4) suggests a lack of contemporary gene flow over this sea. Hence, neither adults nor pelagic larvae seem presently able to successfully enter Scandinavian populations from the south. This lack of dispersal ability is likely a result of the species' preference for rocky substrate, which is completely lacking along the shoreline connecting Scandinavia and mainland Europe, i.e. the western coasts of Denmark, Germany and the Netherlands. Furthermore, the oceanographic modelling strongly suggested that the relatively short pelagic larval duration of the species is too brief for successful larval transport over the long maritime distance into the Scandinavian coasts. These considerations indicate that the original colonization of Scandinavian shores may have taken place at a period when conditions where different from today. Indeed, geological and archaeological evidence have demonstrated that what is presently a shallow ocean (the North Sea) from about 20000 to 7500 BP was dry land (dubbed Doggerland: [53], [54]). This land once represented a continuous shoreline, connecting present day UK with the eastern (Swedish) Skagerrak coast, and may have provided a suitable rocky substrate for the corkwing wrasse to nest underway on its northward range shift towards Scandinavia. Such a possible dispersal route via northern UK is favoured by our assignment analyses (Table 6), which indicated a closer relationship with Scandinavian populations to the northern UK, over the alternative “sandy” southern route through the English channel proposed for several other inshore species [13], [52], [55].

The finding of higher genetic divergence across open water, (i.e. discontinuous habitat) than along rocky shoreline, has been documented for several other coastal marine fish with limited pelagic larval stage and specific habitat requirements (e.g. anemonefish: [56]; blennies: [57], [58], coral fishes: [59]). Interestingly, when comparing our results to those reported for *Taurulus bubalis* [60], a coastal species with similar life history and latitudinal range distribution, we found a similar level of differentiation across the North Sea. However, Scandinavian populations of *S. melops* showed a marked reduction in levels of genetic variability, contrasting with *T. bubalis* which displayed homogenous high levels throughout its range. Almada et al. [60] estimated that northern populations of *T. bubalis* had an older origin than the one predicted for corkwing wrasse [20], suggesting an earlier northward expansion just after the LGM. The high levels of genetic diversity observed in the northern populations of that species may have resulted from a high influx of southern migrants over a long time period [60].

### Conclusion

The finding that the corkwing wrasse to the north of the UK are genetically highly different (and apparently completely isolated) from their southern conspecifics, has important implications for our understanding of the colonization processes and range shift mechanisms following global climate changes. The present isolation of Scandinavian populations implies that the historical range-shift associated with its post-glacial colonization was a unique event, most likely made possible by the presence of suitable shorelines along Doggerland that temporally acted as a migration path from UK to the Skagerrak shores. Following the subsequent submergence of Doggerland the species was apparently deprived of this northward path, and migration to Skagerrak ceased. As judged by the reduced genetic variability in present corkwing wrasse populations in the Skagerrak, the number of successful migrants may anyway have been limited. This scenario implies that species’ range-shifts may be a complicated event, even in the oceans, whose continuous expanse have traditionally been regarded as highly conductive to animal dispersal [8]. Instead, species with specific habitat requirements may be restricted in their ability to response to climate change by a shift in range. The current increase in the species abundance in Scandinavia is not a result of immigration from south, which would have rapidly wiped out genetic differences, but by increased growth of local populations, most likely because rising sea temperatures in this northern range are favourable to the species’ requirements. While the corkwing wrasse may find suitable rocky shores to continue its shift further north as water temperatures continue to rise in the coming decades, they bring with them only a part of the species’ original genetic variability and the loss could impair its long-term survival in a changing environment. Because other species have shown a similar pattern of increased genetic divergence and reduced dispersal among habitable areas (references above), the findings of the present study could represent a more general phenomenon indicating that range-shift may not always be a viable response to climate change.
